# Case Series of Vaccine Hesitancy Encountered at the Pediatric Emergency Department

**DOI:** 10.7759/cureus.74777

**Published:** 2024-11-29

**Authors:** Kensuke Shoji, Yoshiko Uchida, Satoko Uematsu, Tatsuki Ikuse, Isao Miyairi

**Affiliations:** 1 Division of Infectious Diseases, National Center for Child Health and Development, Tokyo, JPN; 2 Department of Emergency and Transport Medicine, National Center for Child Health and Development, Tokyo, JPN; 3 Department of Pediatrics, Hamamatsu University School of Medicine, Hamamatsu-shi, JPN

**Keywords:** emergency department, immunization, vaccination hesitancy, vaccine education, vaccine-preventable diseases

## Abstract

Vaccine hesitancy (VH) is a significant concern, although its specific characteristics remain unclear. Moreover, strategies to shift vaccine-hesitant parents' attitudes toward immunization are not yet well-defined. Pediatric emergency department (ED) physicians frequently encounter patients who are inadequately vaccinated due to parental VH; however, it is challenging to allocate sufficient time during ED visits to provide comprehensive vaccination education. Our institution operates a dedicated outpatient clinic that provides vaccine education for parents with vaccine hesitancy identified in the pediatric ED. The aim of this study was to identify the characteristics of vaccine-hesitant individuals presenting to a pediatric ED in Japan and to assess the effectiveness of our vaccination education clinic.

We found 78 vaccine hesitancy cases in the patients who visited the pediatric ED. Among them, 31 (40%) visited our vaccination education clinic. The majority (29/31, 94%) of parents who attended our vaccination education clinic replied that they gained a positive view of vaccination. The pediatric ED is a useful location to identify VH patients and parents. Vaccination education by pediatric infectious diseases specialists may be useful in changing parental attitudes towards immunization.

## Introduction

Vaccine hesitancy (VH) is a global public health threat [1]. A decline in vaccination rates is known to lead to an increase in the incidence of the infections covered by those vaccines. For example, in the United States, a 5% decrease in the measles-mumps-rubella (MMR) vaccination rate was reported to have resulted in a threefold increase in the number of measles cases among children aged two to 11 with an additional 2.1 million US dollars in public sector costs [2]. VH is also an important issue in Japan, although the characteristics of VH are unknown. In addition, the tactics to change vaccination-hesitant parents’ attitudes towards immunization are still unclear.

Emergency department (ED) practitioners often encounter patients who have not received adequate vaccinations due to hesitant parents [3]; however, it is difficult to secure enough time in the ED to perform education regarding vaccinations during ED visits.

The National Center for Child Health and Development, a tertiary children’s hospital in Japan, initiated cooperation between the ED and the pediatric infectious diseases (ID) division to counter VH. ED physicians inquire about vaccination history to assess the immune status of patients, especially in the context of infectious diseases. This helps guide appropriate treatment and isolation protocols. Additionally, for trauma patients, knowledge of vaccination history is crucial for determining the need for tetanus prophylaxis. Although it may seem time-consuming, this practice is part of routine clinical care that contributes to patient safety and informs critical decision-making, ultimately improving patient outcomes. When ED physicians identify VH parents, they offer them an appointment at a vaccination education clinic run by pediatric ID specialists. 

The purpose of this study was to reveal the characteristics of the parents/guardians with VH who were found at a pediatric ED in Japan and evaluate the utility of our vaccination education clinic.

This study was presented in part at the AAP National Conference and Exhibition, October 2020 and the abstract is available [4].

## Materials and methods

Study design, setting, and population

This study is a case series of vaccine-hesitant patients/parents who presented to a pediatric ED from March 2018 to July 2023. As part of routine work in the ED, physicians review vaccination records by checking the Japanese Maternal and Child Health (MCH) Handbook. The Japanese MCH Handbook is provided to expectant mothers and functions as a comprehensive record-keeping tool. It consolidates health information, advice, and educational resources for both the mother and child, covering the periods of pregnancy, childbirth, and postpartum. This includes details on maternal health care, the child's development, and vaccination schedules [5]. Most parents carry this handbook upon health visitations and the vaccine records can be viewed in two pages of this book. When records were unavailable, parents were briefly questioned whether vaccines were up to date. VH was identified when ED physicians judged that the patient's vaccination schedule was delayed compared to the national standard schedule. If ED physicians noticed that a child was significantly behind the national standard vaccination schedule, they suspected VH among the parents or guardians and informed them about the availability of a vaccination education outpatient clinic. Physicians could also make an appointment for this clinic immediately upon request.

Study measures

A retrospective chart review of the patients was performed. All patients who were found to be VH at the ED and who had an appointment at our vaccine education clinic were reviewed. Patient characteristics, including age, sex, reasons for the VH, and outcomes of the vaccination education clinic, were extracted from the electronic medical records. At the vaccine education clinic, pediatric ID physicians address parental concerns and provide thorough evidence regarding the pros and cons of vaccinations, averaging 30 minutes to one hour per session. At the end of the session, the ID physician asked the parents if attending the vaccine clinic had given them a more positive view of vaccination. The categorical and continuous values were summarized as the number (%) and median (Interquartile range: IQR), respectively. In order to reflect the parents' opinion, we described the comments from parents who visited the vaccination education clinic.

Ethics statement

The study was approved by the ethics committee at the National Center for Child Health and Development (NCCHD-2185 and 2023-101).

## Results

During the study period, 119,369 patients under 15 years of age visited our pediatric ED. Among these, 78 patients/parents were found to be hesitant to receive vaccinations. The patient characteristics are summarized in Table 1.

**Table 1:** Patient characteristics IQR, interquartile range ^#^When multiple reasons were identified, the most clinically significant one was selected. ^*^Lost opportunity to visit a clinic for vaccination; Concern for contracting other infectious diseases when visiting a clinic for vaccination; Religious reasons of a relative; Under consideration

N=78	Median or Number	IQR or %
Age (years)	2.2	1.0-4.4
Male sex	42	53.8%
Reason for the emergency department visit		
Medical (fever, seizure, etc.)	63	80.8%
Surgical (including trauma and orthopedics, etc.)	14	17.9%
Unknown	1	1.3%
Vaccination status		
No vaccination	48	61.5%
Partial vaccination	28	35.9%
Unknown status	2	2.6%
The main reason for vaccination hesitancy^#^		
Concern for adverse events	63	80.8%
Do not feel the necessity of vaccination	4	5.1%
Opinion by primary doctors against immunization	2	2.6%
Others*	4	5.1%
Unknown	5	6.4%
Who in the family holds a vaccine-hesitant opinion?		
Mother and Father	42	53.8%
Father alone	6	7.7%
Mother alone	20	25.6%
Unknown	10	12.8%
Wishes to consult with a pediatric infectious disease specialist regarding immunizations		
Yes	56	71.8%
No	20	25.6%
Unknown	2	2.6%

The median (IQR) age was 2.2 (1.0-4.4) years and 42 (53.8%) were male. The majority (81%) of patients visited ED for medical reasons (fever, seizure, etc.). Forty-eight (62%) of patients did not receive any vaccine. In about half of the cases (42/78, 53.8%), the parents' opinions were in agreement, but in some cases, there was a divergence in the parents' opinions. The main reason for VH was concern about side effects. Ultimately, 31/78 (39.7%) patients visited the vaccination education clinic. The majority (29/31, 93.5%) of parents who attended our vaccination education clinic replied that they gained a positive view of vaccination. Actual comments from the parents are summarized in Table 2.

**Table 2:** Comments from parents who visited the vaccination education clinic

Comments
We felt that there was insufficient opportunity to learn and obtain information about immunization.
It was hard to choose between the opposing opinions of those who were for and against immunization.
We were surprised to hear that chicken pox or mumps could be serious.
We could not accept the guidance from our primary care doctor who said “You should immunize your child without giving it any thought.”
We were threatened multiple times by people around us who pressured us to vaccinate our child.
Are there social disadvantages or repercussions of not getting vaccinated?
We would like to receive as few vaccines as possible. Which ones should we prioritize?

## Discussion

The frequency of VH varies according to regions or settings. Several reports from Western countries indicate the presence of a notable proportion of individuals with VH. For example, a telephone survey conducted in the United States among parents of children aged six to 23 months found that 3.4% of parents refused all vaccines [6]. In a study from Washington State, 9.7% of parents were vaccine-hesitant at the time of their child's birth [7]. Additionally, a survey in European countries revealed that 11.9-27.6% of parents expressed some level of doubt or concern regarding vaccines [8]. In our study, we found 78 cases of vaccine-hesitant parents but we could not accurately estimate the frequency of VH in our research design because it was difficult to rule out the possibility of underreporting due to the lack of objective criteria for defining VH. Therefore, it is necessary to create objective criteria for reporting VH and to estimate the exact frequency of VH in ED in the future.

Our study showed that approximately 40% of VH patients/parents visited the vaccine education clinic. VH parents represent a heterogenous group ranging from those with strong convictions against vaccination to those who are somewhat reluctant. It is well known that there are many reasons for VH, including concerns about safety, efficacy, and freedom of choice about immunization [9]. In our study, the most common reason for VH among those who visited our clinic was parental concerns about vaccine safety. This indicates the importance of relieving vague anxiety about vaccine safety held by parents by providing comprehensive explanations from medical staff.

In our study, more than 90% of parents who attended our vaccination education clinic answered that they gained a positive view of vaccination after the session. It has been reported that the most important factor for parental acceptance of vaccination was communication from the pediatrician [10]. It was also reported that a multifaceted approach including education was useful to increase the vaccination rate of children with VH parents [11]. In our study, concerns about adverse reactions accounted for the majority of reasons for VH. Considering that most parents who visited our vaccine clinic became more willing to proceed with vaccination, providing thorough and evidence-based explanations about vaccine adverse reactions appears to be an effective approach to alleviating such concerns. In addition, some of the parents who visited our clinic expressed a lack of opportunity to learn about immunization. This suggests that there is an underlying demand for an accommodating clinic that provides thorough education for VH parents. VH has been recognized as a critical public health issue, as it has been associated with decreased vaccination coverage and subsequent increases in the incidence of vaccine-preventable diseases [1,2]. In this context, studies such as the present research, which explore strategies to encourage vaccination among vaccine-hesitant parents, are considered essential for addressing this challenge effectively. On the other hand, there was a small but significant number of patients who received recommendations against vaccination from their primary care provider. Whereas it is unclear as to whether patients sought providers based on prior knowledge of such homeopathic clinics, it represents a growing concern that needs to be confronted at a community level.

This study has several limitations. First, we did not use any specific criteria to identify the VH cases. ED physicians who were instructed to check vaccination records were identified as VH if they checked the vaccination record and determined that the vaccination was delayed compared to the standard vaccination schedule. It would be desirable to identify VH using more objective criteria in the future. Second, it did not clearly identify the factors that influenced vaccine-hesitant parents to reconsider vaccination. Recent studies suggest that Motivational Interviewing (MI) may be an effective approach to changing the attitudes of vaccine-hesitant parents [12]. Future research should explore various methods, including MI, to better understand the factors that can help mitigate VH. Last, this study did not track whether the patients ultimately received the vaccines following their visit to the vaccination clinic. Future studies should be performed to confirm whether vaccination was actually administered.

## Conclusions

We found 78 patients/parents who were hesitant to receive vaccinations. We believe the ED is one of the useful places to identify VH patients/parents. The majority of parents who joined the vaccination education clinic obtained a positive view of vaccination. Therefore, vaccination education by pediatric ID specialists may be useful in changing parental attitudes towards immunization. Further investigation is required to confirm whether patients actually received vaccination after attending the clinic.
